# Pathway Compartmentalization in Peroxisome of *Saccharomyces cerevisiae* to Produce Versatile Medium Chain Fatty Alcohols

**DOI:** 10.1038/srep26884

**Published:** 2016-05-27

**Authors:** Jiayuan Sheng, Joseph Stevens, Xueyang Feng

**Affiliations:** 1 Department of Biological Systems Engineering, Virginia Polytechnic Institute and State University, Blacksburg, VA 24061, USA.

## Abstract

Fatty alcohols are value-added chemicals and important components of a variety of industries, which have a >3 billion-dollar global market annually. Long chain fatty alcohols (>C12) are mainly used in surfactants, lubricants, detergents, pharmaceuticals and cosmetics while medium chain fatty alcohols (C6–C12) could be used as diesel-like biofuels. Microbial production of fatty alcohols from renewable feedstock stands as a promising strategy to enable sustainable supply of fatty alcohols. In this study, we report, for the first time, that medium chain fatty alcohols could be produced in yeast via targeted expression of a fatty acyl-CoA reductase (TaFAR) in the peroxisome of *Saccharomyces cerevisiae*. By tagging TaFAR enzyme with peroxisomal targeting signal peptides, the TaFAR could be compartmentalized into the matrix of the peroxisome to hijack the medium chain fatty acyl-CoA generated from the beta-oxidation pathway and convert them to versatile medium chain fatty alcohols (C10 & C12). The overexpression of genes encoding PEX7 and acetyl-CoA carboxylase further improved fatty alcohol production by 1.4-fold. After medium optimization in fed-batch fermentation using glucose as the sole carbon source, fatty alcohols were produced at 1.3 g/L, including 6.9% 1-decanol, 27.5% 1-dodecanol, 2.9% 1-tetradecanol and 62.7% 1-hexadecanol. This work revealed that peroxisome could be engineered as a compartmentalized organelle for producing fatty acid-derived chemicals in *S. cerevisiae*.

As an important raw chemical material, fatty alcohols have been widely used to produce detergents, emulsifiers, lubricants, cosmetics, and fuels^1^. In 2006, over 1.3 million tons of fatty alcohols were used worldwide each year^2^. As a whole, the industry represents a >3-billion-dollar market. The majority of the fatty alcohol market targets on medium chain (C6-C12) or short chain fatty alcohols (<C6). Currently, fatty alcohols are mainly produced in two ways. One is direct extraction from natural plant oils^3^, and the other is chemical synthesis from petrochemical sources. These methods have limitations due to the competition with the food supply^4^ and generation of environmental concerns^5^.

Recently, with the development of metabolic engineering, microbial production of fatty alcohols from renewable feedstock has been achieved successfully in both *Escherichia coli*^2^,^6^ and *Saccharomyces cerevisiae*^7^,^8^. In *E. coli*, fatty alcohols have been produced by introducing heterologous enzymes such as carboxylic acid reductase (CAR)^9^, a bifunctional fatty acyl-CoA reductase (FAR)^10^ or combined expressing an fatty aldehyde-forming fatty acyl-CoA reductase (ACR) with the endogenous aldehyde reductases (AR)^2^,^11^. Recent research showed that a high level of 1-dodecanol and 1-tetradecanol could be produced in *E. coli* by overexpression an acyl-ACP thioesterase (BTE), an acyl-CoA ligase (FadD), and an acyl-CoA/aldehyde reductase (MAACR)^2^. Compared to *E. coli*, yeast, especially *S. cerevisiae*, is also a well characterized and robust industrial host but can be cultivated at lower pH and various harsh fermentation conditions^12^. In addition, *S. cerevisiae* uses type I fatty acids synthetase (FAS) rather than type II FAS as that used by *E. coli*. Compared to that in *E. coli*, the synthetic route of fatty acyl-CoA is shorter in yeast, which could allow more efficient conversion of carbohydrate substrates to fatty acids and fatty acid-derived biofuels^6^. Therefore, there has been an increasing interest on developing yeast, such as *S. cerevisiae*, as a cell factory for production of fatty acid-derived chemicals. In *S. cerevisiae*, a mouse FAR has been expressed to produce 1-hexadecanol. Through over-expression of acetyl-CoA carboxylase (ACC1) and fatty acid synthases (FAS1 and FAS2), the engineered *S. cerevisiae* strain produced 98.0 mg/L 1-hexadecanol from 20 g/L hexose in batch fermentation in minimal medium^7^. In our previous study, we found that by manipulating the structural genes in yeast lipid metabolism, tuning the regulation of phospholipid synthesis, and increasing the supply of key precursors, 1-hexadecanol was produced at 1.1 g/L as the sole fatty alcohol product using glucose as the sole carbon source in a fed-batch fermentation^8^.

Despite the success on engineering *S. cerevisiae* to produce long chain fatty alcohols, no success, to our best knowledge, has been achieved to engineer *S. cerevisiae* to produce medium chain fatty alcohols. This is mainly because of the lack of the precursor (i.e., medium chain fatty acyl-CoA) for producing medium chain fatty alcohols in *S. cerevisiae*, since the majority of fatty acyl-CoAs in native *S. cerevisiae* have long chains^13^. Recently, several pioneering studies have engineered *S. cerevisiae* to produce medium and short chain fatty acids by replacing the native type I FAS with heterologous type II FAS^14^ or disrupting the beta-oxidation^15^,^16^. However, the titers of medium and short chain fatty acids in these engineered yeast strains were still too low (<200 mg/L) to meet the demands of fatty acid-derived chemicals such as medium chain fatty alcohols.

In this study, we developed a novel strategy to engineer *S. cerevisiae* to produce medium chain fatty alcohols by compartmentalizing the fatty alcohol synthesis pathway in peroxisome. In native *S. cerevisiae*, the pool of shortened fatty acyl-CoAs only exist in peroxisome as the intermediates of the beta-oxidation cycle, which catalyzes the chain shortening of fatty acyl-CoA esters between carbons 2 and 3, yielding products as chain-shortened acyl-CoA and acetyl-CoA or propionyl-CoA, depending on substrates^17^. We hence assumed that by expressing TaFAR in peroxisome, TaFAR could hijack the chain-shortened acyl-CoAs to produce medium or even short chain fatty alcohols (Fig. 1). Indeed, when we introduced a series of peroxisomal targeting signal peptide to import TaFAR into peroxisome, versatile medium chain fatty alcohols, including 1-decanol, 1-dodecanol and 1-tetradecanol, could be detected in the engineered *S. cerevisiae* strains. We then manipulated the structural proteins in peroxisome (e.g., PEX7) to enhance the efficiency of enzyme location into peroxisome and overexpressed ACC1 to overcome the rate limiting steps in fatty acid synthesis. Over 1.3 g/L medium chain and long chain fatty alcohols were produced in total in a fed-batch fermentation using an optimized medium, which represents the highest titer of fatty alcohol reported in *S. cerevisiae* so far. To our best knowledge, it is first time that yeast was engineered to produce medium chain fatty alcohols. Our work demonstrated that the compartmentalization of metabolic pathways stands as a promising strategy to produce fatty acid-derived chemicals.

## Results

### Using PTS signaling pathway for targeted expression of FAR in peroxisome

In *S. cerevisiae*, the cytosol type I FAS, which is a highly integrated multi-enzyme complex, mainly produces long chain fatty acyl-CoA (e.g., C16:0) in the cell. Therefore, when expressing a FAR gene, such as TaFAR from barn owl *Tyto alba*, in yeast, the produced fatty alcohol was mainly in the form of 1-hexadecanol^8^. To produce medium chain fatty alcohols, the shorter chain fatty acyl-CoAs are required as the precursors. The shortened fatty acyl-CoAs could be generated in peroxisome as important intermediates in the beta-oxidation cycle that degrades fatty acids and exclusively takes place in peroxisomes of fungi and plants^18^. In this case, we assumed that when TaFAR was introduced into peroxisomes, it could use the shortened acyl-CoAs as the precursors to produce medium chain fatty alcohols.

The targeted expression of proteins in peroxisomes requires signal sequences known as peroxisomal targeting signal (PTS). Two types of PTS have been discovered: type I (PTS1) and type II (PTS2)^19^. The PTS1, used by the majority of peroxisomal matrix proteins, is located at the extreme C-terminus and was initially discovered in firefly luciferase as the tripeptide SKL^20^. The PTS1 generally fits the consensus sequence (S/A/C)-(K/R/H)-(L/M)^21^. The PTS2 is a conserved sequence which is located near the N-terminus of a protein and is comprised in some species within a pre-sequence that is cleaved off after import into the peroxisomal matrix. Sequence comparisons of the signal sequences showed the conserved nonapeptide of PTS2 as (R/K)-(L/V/I)-X_5_-(H/Q)-(L/A/F)^22^.

**Figure 2:**
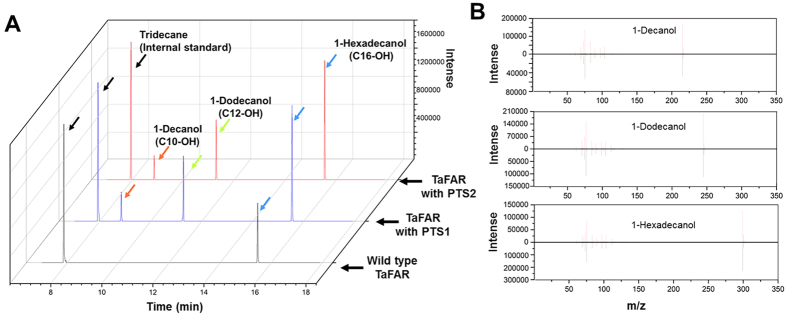
The profile of fatty alcohols produced by the engineered *S. cerevisiae* strains. (**A**) The GC spectrum of fatty alcohols produced by the strains with wild type TaFAR (*S. cerevisiae* INVSc1 with plasmid pFAR) and the TaFAR with PTS1 (*S. cerevisiae* INVSc1 with plasmid pFARPTS01) or PTS2 (*S. cerevisiae* INVSc1 with plasmid pFAR PTS09) peptides. (**B**) The MS spectrum of the fatty alcohols produced by *S. cerevisiae* in comparison with the standards.

To prove the concept that TaFAR can be expressed in peroxisomes of *S. cerevisiae* and produce medium chain fatty alcohols by tagging with a PTS peptide, we selected two PTS peptides: SKL (i.e., a PTS1 peptide) and KL-X5-QL (i.e., a PTS2 peptide), fused them to the C-terminal or N-terminal of TaFAR respectively, and measured the fatty alcohols profiles by GC-MS (Fig. 2). As expected, it was found that both the TaFAR with PTS1 and PTS2 peptides produced long chain and medium chain fatty alcohols (7.7% 1-decanol, 28.3% 1-dodecanol and 64.0% 1-hexadeconal), while the wild type TaFAR only produced 1-hexadeconal (Fig. 2).

**Figure 3:**
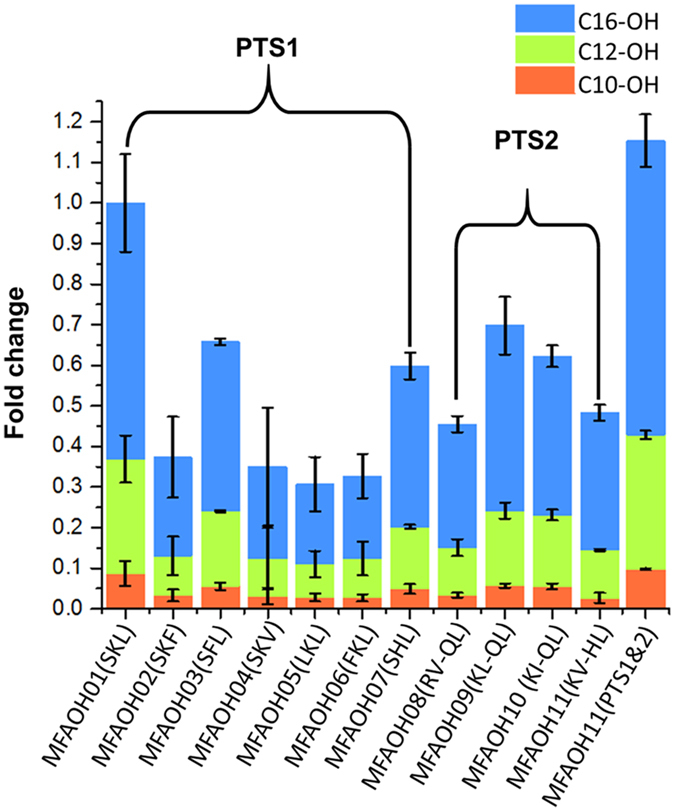
Fatty alcohols produced by engineered *S. cerevisiae* strains via screening PTS signal peptides. All the strains were cultured in batch fermentation with the SC medium for 48 h. The values were all normalized to that of SKL peptide.

**Figure 4:**
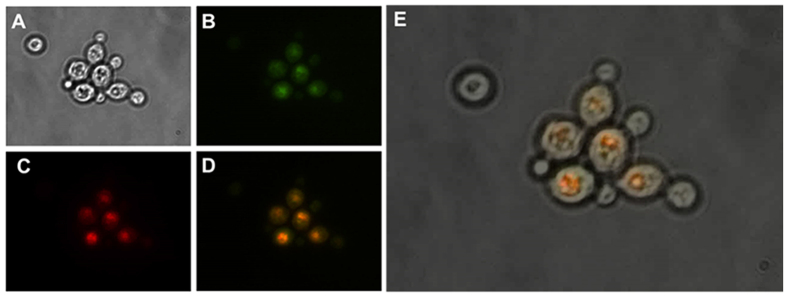
Fluorescence microscopy images of engineered strain PEXFLUO (*S. cerevisiae* BY4741 with plasmids pPEX14GFP, pFARCHERRY) showed the co-localization of the peroxisomal membrane protein PEX14 (fused with GFP), and TaFAR-mCherry tagged with SKL peptide on C-terminal and KL-QL on N-terminal. PEX14 was used as a marker of the peroxisomal matrix. (**A**) The bright field image of *S. cerevisiae* cells. (**B**) The green fluorescence of PEX14 protein fused with GFP. (**C**) The red fluorescence of TaFAR-mCherry protein tagged with SKL and KL-QL. (**D**) The merged image of green fluorescence and red fluorescence. (**E**) The merged image of both fluorescence and bright field. The overlapped green fluorescence and red fluorescence indicated the same location of PEX14 and TaFAR-mCherry, which confirmed the targeted expression of TaFAR in peroxisomes.

In order to select the PTS peptide with the highest efficiency of targeted expression of TaFAR, we next screened 7 PTS1 peptides (-SKL, -SKF -SFL, SKV, -LKL, -FKL, -SHL) and 4 PTS2 peptides (RV-X5-QL, KL-X5-QL, KI-X5-QL, KV-X5-HL), and evaluated the fatty alcohols production by GC-MS (Fig. 3). We found that all of the PTS peptides could lead to the production of medium chain fatty alcohols. Among the PTS1 peptides, TaFAR with SKL had the highest total fatty alcohols production, while among the PTS2 signals KL-QL led to the highest titers of fatty alcohols. We also fused both SKL and KL-X5-QL with TaFAR in C- and N- terminals. Such “double PTS peptide” led to a further 15% improvement of the total fatty alcohols production (Fig. 3). In addition, we used the fluorescence microscopy and confirmed that the “double PTS peptide”, in which TaFAR was tagged with SKL peptide on C-terminal and KL-X5-QL on N-terminal, was indeed expressed in the peroxisome (Fig. 4).

### Engineer the cargo and structural protein in peroxisome to enhance the production of medium chain fatty alcohols

Encouraged by the success on engineering *S. cerevisiae* to produce medium chain fatty alcohols, we next aimed to systemically engineer the cargo and structural proteins of peroxisome to improve the fatty alcohol production. In general, peroxisomal matrix proteins synthesized in the cytosol are bound by peroxisomal receptors – PEX5^23^ in the case of PTS1, and PEX7^24^ in the case of PTS2. These receptor–cargo complexes then move to the peroxisome membrane where they dock with protein sub-complexes that are in or on the membrane. In addition to PEX5 and PEX 7, PEX3 and PEX19^25^ are also essential for peroxisome biogenesis. PEX19 binds newly synthesized peroxisomal membrane proteins post-translationally and directs them to peroxisomes by engaging PEX3, a protein anchored in the peroxisome membrane^26^. Working together, PEX3 and PEX19 mediate the import of membrane proteins as well as the *de novo* formation of peroxisomes. Therefore, overexpression of PEX5, PEX7, PEX3 and PEX19 was hypothesized to enhance the targeted expression of TaFAR and thus increase the titer of fatty alcohols.

**Figure 5:**
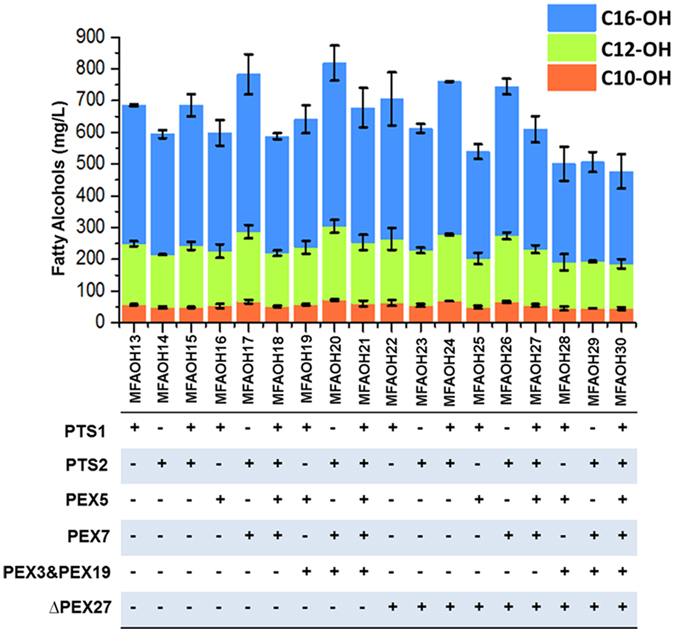
Fatty alcohols produced by engineered *S. cerevisiae* strains via modification of peroxisomal proteins. All the strains were cultured in batch fermentation with the SC medium for 48 h.

To evaluate the effects of PEX5, PEX7, PEX3 and PEX19 on production of medium chain fatty alcohols, we chose SKL and KL-QL as our model PTS1 and PTS2 peptides since they showed the most positive effects on producing medium chain fatty alcohols, and tested different combinations of PTS peptides and overexpression of PEX proteins in batch fermentation. We found that the overexpression of PEX5 with PTS1 peptide could not improve fatty alcohol production, while on the other hand, the overexpression of PEX7 with PTS2 peptide increased fatty alcohol titer by 37.1%, reaching 782.7 ± 77.6 mg/L. The highest fatty alcohol titer was achieved in batch fermentation at 817.9 ± 39.1 mg/L when TaFAR was fused with PTS2 peptide, and PEX7, PEX3, and PEX 19 were overexpressed simultaneously (Fig. 5). Considering the similar titer of the two constructs (i.e., PTS2 with overexpression of PEX7, and PTS2 with overexpression of PEX7, PEX3, PEX19), we chose the strain that overexpressed TaFAR fused with PTS2 and its receptor PEX7 as our target strain for further engineering to avoid the possible high metabolic burdens caused by overexpression of multiple proteins. We also evaluated the effects of knocking out a peripheral peroxisomal membrane protein PEX27^27^, which is a negative regulator in controlling peroxisome size and number, but did not observe the improvement of fatty alcohol titer. It was also worth noting that while the that PTS1&2 led to higher production of fatty alcohols than PTS1 (Fig. 3) when we screened different PTS peptides in INVSc1 strain, the PTS1&2 tagged TaFAR led to similar amount of fatty alcohols as that of PTS1 tagged TaFAR (Fig. 5) when we expressed the same plasmids in BY4741 strain. The different genetic background of *S. cerevisiae* INVSc1 and BY4741 strains could be the reason for such conflict.

### Host engineering to enhance the production of fatty alcohols

**Figure 6:**
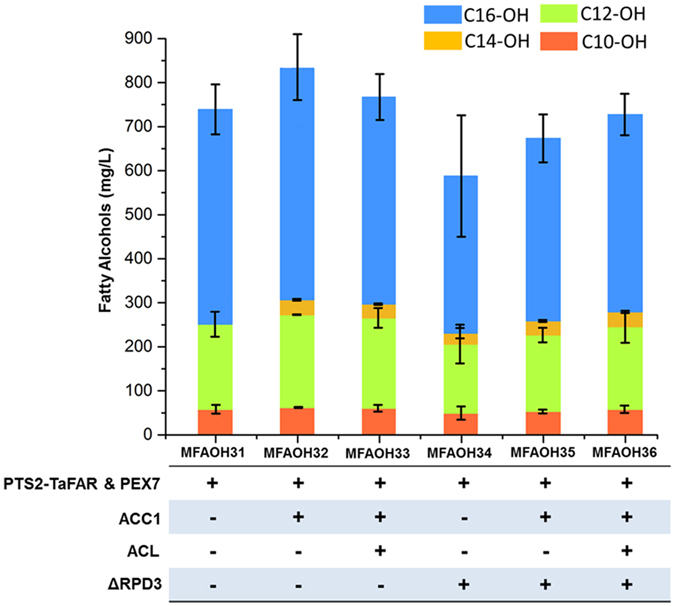
Fatty alcohols produced by engineered *S. cerevisiae* strains via host engineering. All the strains were cultured in batch fermentation with the SC medium for 48 h.

In our previous studies^8^ on engineering *S. cerevisiae* to produce 1-hexadecanol in cytosol, we found that the highest titer of fatty alcohol was achieved when using the strategy of overexpressing ACC1 (a well-known bottleneck in fatty acid synthesis), knocking out RPD3 (a negative regulator for phospholipid synthesis), and introducing a heterologous ATP-dependent citrate lyase (ACL) to over-produce cytosolic acetyl-CoA. Inspired by the previous success on host engineering to enhance the production of long chain fatty alcohols, we used the same strategies to try to increase the production of medium chain fatty alcohols. We chose the strain expressing PEX7 and PTS2-TaFAR as our starting point and comprehensively evaluate the effects of overexpressing ACC1, knocking out RPD3, and the heterologous expression of ACL (Fig. 6). We found that overexpressing ACC1 could slightly increased the production of fatty alcohols, leading to the highest titer at 832.7 ± 73 mg/L. However, knocking out RPD3 and heterologous expression of ACL did not have beneficial effects on improving the fatty alcohol production. Interestingly, we could detect small amount of 1-tetradecanol (<3% of the total fatty alcohol) when engineering ACC1, RPD3 and ACL. As shown in the GC spectrum in Figure S1, A tiny peak of 1-tetradecanol could be detected in the strain with ACC1 over-expressed. However, no match of 1-tetradecanol could be found in the strain without overexpression of ACC1 when searching against the NIST MS library. Overall, the host engineering clearly indicated the shift of the bottlenecks for fatty alcohol production when compartmentalizing pathways in peroxisome.

### Fed-batch fermentation for fatty alcohol production

With the strain overexpressing TaFAR tagged with a PTS peptide, PEX7 and ACC1 as our best strain (MFAOH37) to produce fatty alcohols, we next characterized the fatty alcohol production in fed-batch fermentation with different media. We identified two parameters (C/N ratio and K^+^ ions) that could potentially affect the fatty alcohol production based on previous reports^28^,^29^. In general, it has been found that the production of fatty acid and the derived chemicals could be enhanced^30^ at high C/N ratio (i.e., nitrogen limited). When K^+^ ions were added in the form of KOH and KCl to the culture medium, it was reported the change on the elemental balance could significantly enhance product accumulation in *S. cerevisiae*^28^. Therefore, we designed and tested four media: 1) normal SC medium with C/N ratio at 3:1; 2) nitrogen limited medium with C/N ratio at 50:1; 3) normal SC medium (C/N = 3:1) with K^+^ (a mixture of 40 mM KCl and 10 mM KOH); and 4) nitrogen limited medium (C/N = 50:1) with K^+^ (a mixture of 40 mM KCl and 10 mM KOH).

**Figure 7:**
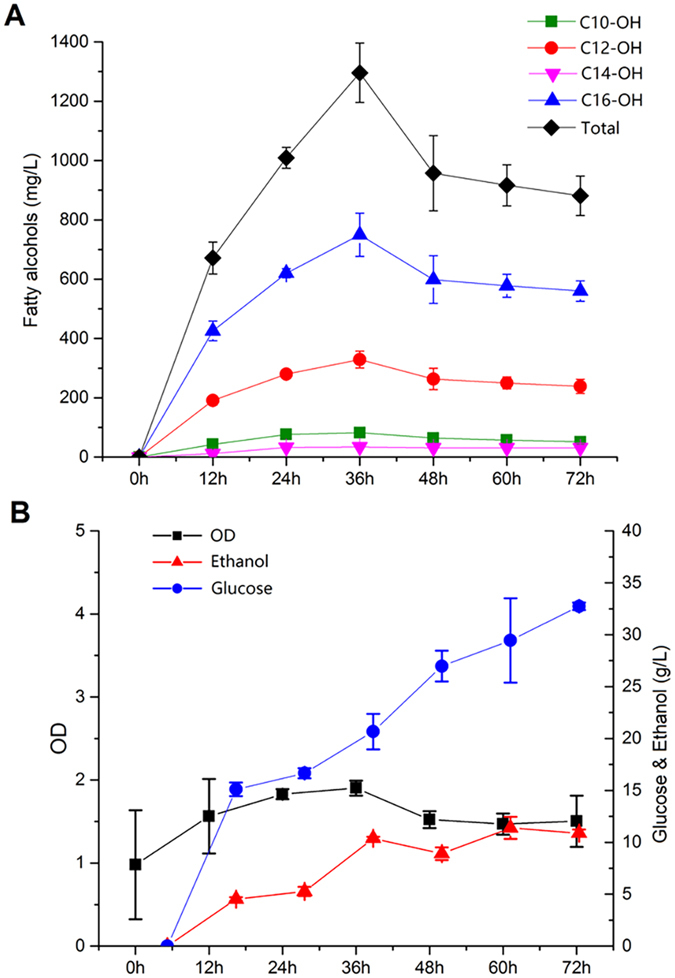
Fed-batch fermentation of strain MFAOH37 (*S. cerevisiae* INVSc1 with plasmids pFARPEX and pACC1) using the nitrogen limited (C:N = 50:1) SC medium. (**A**) The production of fatty alcohols. (**B**) Sugar consumption and byproduct production. Black square: OD_600_; Blue circle: the total glucose consumed; Red triangle: The ethanol produced.

In fed-batch fermentation, we used a resting cell fermentation, in which the cells had marginal net growth rate but rather served as biocatalysts to convert glucose to fatty alcohols. We found that the titer of fatty alcohols using the normal SC medium was 0.98 ± 0.07 g/L; the titer of fatty alcohols using the normal SC medium with K^+^ supplement was 1.11 ± 0.07 g/L and the titer of fatty alcohols using the nitrogen limited SC medium with K^+^ supplement was 1.02 ± 0.08 g/L (Figure S2). The nitrogen limited medium without K^+^ supplied in the fed batch fermentation led to the highest the titer of total fatty alcohols at 1.30 ± 0.10 g/L with 6.9% 1-decanol, 27.5% 1-dodecanol, 2.9% 1-tetradecanol and 62.7% 1-hexadecanol, which represents the highest fatty alcohol titer reported in *S. cerevisiae* (Fig. 7). However, a drop of fatty alcohol concentration was observed together with the OD decrease at 36 h (Fig. 7), which was likely due to the starvation caused by nutrient limitation. The fermentation results confirmed the positive effects of nitrogen limitation and K^+^ supplement on fatty alcohol production in *S. cerevisiae*. Nevertheless, no synergistic effects were found between nitrogen limitation and K^+^ supplement.

## Discussion

Pathway compartmentalization has been recently demonstrated as a novel approach for enhancing the production of a series of chemicals, including isobutanol^31^, isopentanol^31^, 2-methyl-1-butanol^31^, itaconic acid^32^, terpenoids^33^, acetoin^34^, and fatty acids^35^. Compared to the conventional engineering of cytosolic pathways, the compartmentalizing of pathways has several advantages such as favoring faster reaction rates due to the concentrated precursors in the smaller volume of compartments, and avoiding repressive regulatory responses and competing pathways^31^. Till now, most of the studies on pathway compartmentalization focused on mitochondria. In this study, we demonstrated that peroxisome could be another promising candidate to compartmentalize metabolic pathways for synthesis of novel chemicals, especially fatty acid-derived chemicals such as medium chain fatty alcohols, considering the abundance and versatility of fatty acyl-CoA generated from the beta-oxidation in peroxisome. Another unique advantage of using peroxisome for pathway compartmentalization is the easiness in manipulating the peroxisome as a whole. As discovered in this study, the modification of PTS peptides as well as cargo and structural peroxisomal proteins led to the most significant improvement of medium chain fatty alcohols production (from 0 mg/L to ~800 mg/L). On the other hand, the conventional host engineering strategy only marginally improved the peroxisomal production of fatty alcohol. This indicated a paradigm shift when adopting the strategy of pathway compartmentalization with a new set of bottlenecks arising. Compared to the study on functionality of cytosolic pathways, our understanding on peroxisomal pathways is still limited. However, with the in-depth analysis using systems biology approaches (e.g., -omics analysis), we can envision a much better mechanistic understanding on peroxisomal pathways and use such knowledge to further improve the biochemical production in peroxisome.

During the engineering of peroxisomal pathways, we found that 1-hexadecanol was still produced as the main products (>60%) despite of targeted expression of TaFAR. This was not unexpected since the main fatty acid produced by *S. cerevisiae* was palmitic acid, which could generate palmitoyl-CoA as the most abundant fatty acyl-CoA in peroxisome. Therefore, when TaFAR was expressed in peroxisome, it would mainly use palmitoyl-CoA to produce 1-hexadecanol. Interestingly, we could not detect 1-tetradecanol when engineering the PTS peptides or peroxisomal proteins, but observed tiny amount of 1-tetradecanol during host engineering when enhancing fatty acid synthesis. This could be attributed to the fact that the overproduction of fatty acids led to more tetradecanoic acid synthesized, which was further diverted to peroxisome and used as the precursor for 1-tetradecanol production. Also, the substrate specificity of TaFAR could be another reason for the low concentration of 1-tetradecanol. It was worth noting that some other FAR enzymes such as AdFAR1 was reported to prefer medium-chain fatty acyl-CoAs^36^. In fact, as shown in Figure S3, we have characterized the profile of fatty alcohol produced by AdFAR1. In general, we constructed a new pAdFARPEX plasmid in the same way as pFARPEX plasmid, in which the AdFAR was expressed under constitutive TEF1 promoter and tagged with the PTS2 peptide (KL-X5-QL) and the PEX7 was expressed under constitutive TPI1 promoter. The reason that we chose this combination of this PTS2 peptide and PEX7 is that they led to the highest fatty alcohol titer when using TaFAR. By transforming the pAdFARPEX plasmid into BY4741 strain, it was shown that the AdFAR led to the similar titer of fatty alcohols as that of TaFAR (812 ± 92 mg/L in AdFAR and 783 ± 78 mg/L in TaFAR), but different profiles of fatty alcohols (Figure S3). In addition to 1-decanol, 1-dodecanol and 1-hexadecanol, 1-tetradecanol was also produced (8.1% of total fatty alcohols produced) when expressing AdFAR1 and culturing the recombinant yeast in SC medium for 24h in batch fermentation. This indicates by expressing different FARs, the profile of fatty alcohols produced by yeast could vary.

In summary, by compartmentalizing fatty alcohol synthesis pathway in peroxisome, we successfully engineered *S. cerevisiae* to produce medium chain fatty alcohols for the first time. The current production of fatty alcohols reached 1.3 g/L in titer, 36 mg/L/h in productivity, and 43 mg/g in yield. Recently, it was found that when coupled with the enhanced precursor supply, the over-production of NADPH could lead to over five-fold increase of 3-hydroxypropionic acid production^37^. Therefore, it is possible that by coupling with NADPH overproduction and the pathway compartmentalization, the production of fatty alcohols could be further improved. We are currently trying to engineering the cytosolic and peroxisomal NADPH supply, together with our established strategies of targeted expression of TaFAR, overexpressing PEX7 and ACC1 to further increase the production of fatty alcohols.

## Materials and Methods

### Yeast strains, media, and transformation

The yeast strains used in this study were derived from both BY4741 and INVSc1. The cells were stored in a 15% v/v glycerol solution at −80 °C. *E. coli* DH5α strain was used for maintaining and amplifying plasmids and recombinant strains were cultured at 37 °C in Luria-Bertani (LB) broth. Ampicillin at a concentration of 100 μg/mL was added to the LB medium when required. The BY4741 and INVSc1 strains were cultured in YPAD medium. Yeast cells were transformed with plasmids using the LiAc/PEG method as described previously^38^. For selection of the yeast transformants, a synthetic complete (SC) medium was used, which contains 0.17% yeast nitrogen base, 0.5% ammonium sulfate, and the appropriate amino acid dropout mix (MP Biomedicals, Solon, OH). A single colony was picked and cultured in 5 mL SC media containing 20 g/L glucose. The cells were cultured at 30 °C in disposable culture tubes shaken at 250 rpm for 2 days.

### Plasmid construction

An established, yeast homologous recombination-based method, DNA assembler, was used to construct the recombinant plasmids^39^. Basically, DNA fragments sharing homologous regions to adjacent DNA fragments were co-transformed into *S. cerevisiae* along with linearized backbone to assemble several elements in a single step^40^. Oligonucleotides used in this study were listed in Table S1 and the recombinant plasmids constructed in this study were listed in Table 1.

### Fluorescence microscopy for visualizing enzyme localization

To confirm TaFAR was being transported into the peroxisome fluorescence microscopy was used. Two fluorescent proteins were used in the imaging: Green Fluorescent Protein (GFP) and mCherry. To highlight the membrane of the peroxisome, the GFP was fused to PEX14, a protein known to be embedded in the peroxisomal membrane. To highlight the interior of the peroxisome, TaFAR-mCherry was tagged with SKL peptide on C-terminal and KL-X5-QL on N-terminal. Yeast cells were transformed using the methods previously described with the plasmids containing PEX14-GFP and TaFAR-mCherry and grown in 3 mL of SC media containing all appropriate amino acids, with 20 g/L glucose for 2 days. Then, 1 mL of the yeast cells were centrifuged, washed and re-suspended in 1 mL distilled water. 5 μL of the yeast cells were mixed with 10 μL of 1% melted agarose and then overlaid with a glass cover slip to fix the cells in place. To visualize the PEX14-GFP protein, the excite wavelength was 488 nm and emission wavelength was 525 nm. To visualize the TaFAR-mCherry protein, the excitation wavelength was 587 nm and emission wavelength was 610 nm. The overlapped green fluorescence and red fluorescence indicated the same location of PEX14 and TaFAR and confirmed the targeted expression of TaFAR in peroxisomes.

### Measurement of fatty alcohol production

The fatty alcohol production of the engineering yeast cells was screened in triplicate following a published protocol^8^. In general, to screen for the production of fatty alcohols, the engineered yeast strains were pre-cultured in disposable culture tubes with 3 mL of synthetic complete (SC) media with all appropriate amino acids and 2% glucose for three days until saturation. Then, 0.5 mL of the cells were transferred to glass tubes containing 4.5 mL of fresh SC media overlaid with 10% (v/v) dodecane to extract the fatty alcohols. The concentrations of fatty alcohols were quantified at 48 h. The glass tubes of yeast culture were allowed to sit for several minutes until the organic layers were clearly visible. Then, 3 μL of dodecane was extracted from the organic layer and diluted 100 times using ethyl acetate containing 20 mg/L tridecane as the internal standard. Of this mixture, 60 μL was added to 40 μL BSTFA with 1% TMCS solution and incubated at 70 °C for 30 minutes. This solution was centrifuged and analyzed by GC-MS (Shimadzu GC-MS-QP2010) with a DB-Wax column with 0.25 μm film thickness, 0.25 mm diameter, and 30 m length (Agilent Inc., Palo Alto, CA). The GC program was as follows: an initial temperature of 50 °C was maintained for 1.5 min, followed by ramping to 180 °C at a rate of 25 °C/min. The temperature was then ramped to 250 °C at a rate of 10 °C/min, where the temperature was held for 3 min. The final titer of fatty alcohol (mg/L) was calculated as 10 × GC data. All the titers we reported were the results that were adjusted to final titer (i.e., mg/L fatty alcohol produced in the fermenter). Since we were not sure what type of fatty alcohols (e.g., odd chain fatty alcohols) could be produced when expressing FARs in peroxisome, we decided to choose tridecane instead of tridecanol or pentadecanol as our internal standard. We found a very linear correlation between the fatty alcohol concentration (i.e., 1-decanol, 1-dodecanol, 1-tetradecanol and 1-hexadecanol) and the ratio of GC peak area of tridecane to fatty alcohol standards, with the R^2^ reaching 0.90 or higher.

### Medium optimization and fed-batch fermentation

For fed-batch fermentation, the engineered yeast strains were first grown in 40 mL SC media including all appropriate amino acids, with 20 g/L glucose and allowed to grow for 48 h. Then, 25 mL of the cell cultures were centrifuged, washed twice with sterilized water, re-suspended with 5 mL of SC media in glass tubes and overlaid with 10% dodecane to extract the fatty alcohol. The correlation between the OD measurement in our lab and the DCW was found as 1 OD ≈ 4 g/L DCW. Therefore, in the fed-batch fermentation, there were 8~10 g/L DCW. This number (i.e., 8~10 g/L DCW) was still lower than the theoretical biomass yield from glucose (i.e., ~0.5 g/g). This is mainly because of the metabolic burden when expressing multiple plasmids, which was found to slow down the cell growth in this study. Therefore, when we harvested the cells at 48 h from the batch culture, the OD of the cells had not yet reached the saturation. Fermentations were performed in triplicate and tested every 12 h. Fatty alcohol levels were measured using the GC-MS procedure described above, remaining glucose levels and ethanol production were measured by HPLC, and OD was measured using the plate reader. Additional glucose (0.5 mL with concentration of 200 g/L) and dodecane (0.05 mL) were fed every 24 h. To optimize the medium in the fed-batch fermentation, four different types of media were used to evaluate fatty alcohol production: 1) Normal SC medium (control condition with C:N ratio at 3:1), with the glucose concentration at 20 g/L and yeast nitrogen base (without amino acids) concentration at 6.7 g/L; 2) Nitrogen limited SC medium with C:N ratio at 50:1, with the glucose concentration at 20 g/L, and yeast nitrogen base (without amino acids) concentration at 0.4 g/L; 3) Normal SC medium with supplement of 40 mM KCl and 10 mM KOH; and 4) Nitrogen limited SC medium with C:N ratio at 50:1, supplement of 40 mM KCl and 10 mM KOH. Triplicates of each type of medium were performed. The same fed-batch procedure and protocol of fatty alcohol measurement were used as described above.

## Additional Information

**How to cite this article**: Sheng, J. *et al*. Pathway Compartmentalization in Peroxisome of *Saccharomyces cerevisiae* to Produce Versatile Medium Chain Fatty Alcohols. *Sci. Rep*. **6**, 26884; doi: 10.1038/srep26884 (2016).

## Supplementary Material

Supplementary Information

## Figures and Tables

**Figure 1:**
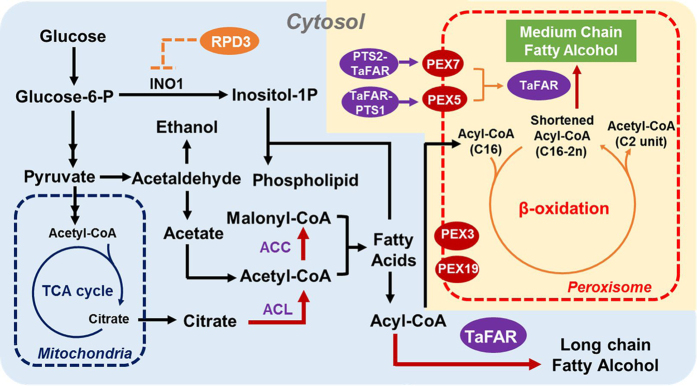
Scheme of engineering yeast peroxisome for medium chain fatty alcohols production. Fatty alcohols can be produced by expressing a fatty acyl-CoA reductase from *Tyto alba* (TaFAR). TaFAR was then introduced into matrix of peroxisome by tagging with PTS1 or PTS2 signals to convert shortened fatty acyl-CoAs to produce medium chain fatty alcohols. The peroxisomal membrane protein PEX5 and PEX7 was overexpressed to enhance the efficiency of cytosol TaFAR introduction. PEX3 and PEX19 were engineered to help the formation of peroxisomes. In addition, acetyl-CoA carboxylase (ACC1) and ATP-dependent citrate lyase (ACL) from *Yarrowi lipolytica* were overexpressed to enhance the supply of precursors in fatty acid synthesis.

**Table 1:** Plasmids and strains used in this study.

Plasmids used in this study			
Name	Description		Reference
pYlACL	pRS423-TPI1p-YlACL1-TPI1t-TEF1p-YlACL2-TEF1t		Lian *et al*.^41^
pFAR	pRS416- TEF1p-TaFAR-TEF1t		This study
pFARPTS01	pRS416- TEF1p-TaFAR-SKL-TEF1t		This study
pFARPTS02	pRS416- TEF1p-TaFAR-SKF-TEF1t		This study
pFARPTS03	pRS416- TEF1p-TaFAR-SFL-TEF1t		This study
pFARPTS04	pRS416- TEF1p-TaFAR-SKV-TEF1t		This study
pFARPTS05	pRS416- TEF1p-TaFAR-LKL-TEF1t		This study
pFARPTS06	pRS416- TEF1p-TaFAR-FKL-TEF1t		This study
pFARPTS07	pRS416- TEF1p-TaFAR-SHL-TEF1t		This study
pFARPTS08	pRS416- TEF1p-RV-X5-QL-TaFAR-TEF1t*		This study
pFARPTS09	pRS416- TEF1p-KL-X5-QL-TaFAR-TEF1t		This study
pFARPTS10	pRS416- TEF1p-KI-X5-QL-TaFAR-TEF1t		This study
pFARPTS11	pRS416- TEF1p-KV-X5-HL-TaFAR-TEF1t		This study
pFARPTS12	pRS416- TEF1p-KV-X5-HL-TaFAR-SKL-TEF1t		This study
pPEX01	pRS415- TEF1p-PEX5-TEF1t		This study
pPEX02	pRS415- TEF1p-PEX7-TEF1t		This study
pPEX03	pRS415- TEF1p-PEX5-TEF1t- TPI1p-PEX7-CYC1t		This study
pPEX04	pRS415- TEF1p-PEX3-TEF1t- TPI1p-PEX19-CYC1t		This study
pACC1	pRS415- TEF1p-ACC1-TEF1t		This study
pFARPEX	pRS416- TEF1p-KL-X5-QL-TaFAR-TEF1t-TPI1p-PEX7-CYC1t		This study
pAdFARPEX	pRS416- TEF1p-KL-X5-QL-AdFAR-TEF1t-TPI1p-PEX7-CYC1t		This study
pPEX14GFP	pRS416- TEF1p-PEX14-GFP-TEF1t		This study
pFARCHERRY	pRS415- TEF1p-KL- X5-QL-TaFAR-mCherry-SKL-TEF1t		This study
**Strains used in this study**			
**Name**	**Phenotype**	**Plasmids**	**Reference**
BY4741	MATa his3Δ1 leu2Δ0 met15Δ0 ura3Δ0	—	This study
BY4741ΔRPD3	MATa his3Δ1 leu2Δ0 met15Δ0 ura3Δ0 Δrpd3	—	This study
BY4741ΔPEX27	MATa his3Δ1 leu2Δ0 met15Δ0 ura3Δ0 Δpex27	—	This study
INVSc1	MATa his3D1 leu2 trp1-289 ura3-52 MAT his3D1 leu2 trp1-289 ura3-52	—	This study
FAOH	Same as INVSc1	pFAR	This study
MFAOH01	Same as INVSc1	pFARPTS01	This study
MFAOH02	Same as INVSc1	pFARPTS02	This study
MFAOH03	Same as INVSc1	pFARPTS03	This study
MFAOH04	Same as INVSc1	pFARPTS04	This study
MFAOH05	Same as INVSc1	pFARPTS05	This study
MFAOH06	Same as INVSc1	pFARPTS06	This study
MFAOH07	Same as INVSc1	pFARPTS07	This study
MFAOH08	Same as INVSc1	pFARPTS08	This study
MFAOH09	Same as INVSc1	pFARPTS09	This study
MFAOH10	Same as INVSc1	pFARPTS10	This study
MFAOH11	Same as INVSc1	pFARPTS11	This study
MFAOH12	Same as INVSc1	pFARPTS12	This study
MFAOH13	Same as BY4741	pFARPTS01	This study
MFAOH14	Same as BY4741	pFARPTS09	This study
MFAOH15	Same as BY4741	pFARPTS12	This study
MFAOH16	Same as BY4741	pFARPTS01, pPEX01	This study
MFAOH17	Same as BY4741	pFARPTS09, pPEX02	This study
MFAOH18	Same as BY4741	pFARPTS12, pPEX03	This study
MFAOH19	Same as BY4741	pFARPTS01, pPEX01, pPEX04	This study
MFAOH20	Same as BY4741	pFARPTS09, pPEX02, pPEX04	This study
MFAOH21	Same as BY4741	pFARPTS12, pPEX03, pPEX04	This study
MFAOH22	Same as BY4741ΔPEX27	pFARPTS01	This study
MFAOH23	Same as BY4741ΔPEX27	pFARPTS09	This study
MFAOH24	Same as BY4741ΔPEX27	pFARPTS12	This study
MFAOH25	Same as BY4741ΔPEX27	pFARPTS01, pPEX01	This study
MFAOH26	Same as BY4741ΔPEX27	pFARPTS09, pPEX02	This study
MFAOH27	Same as BY4741ΔPEX27	pFARPTS12, pPEX03	This study
MFAOH28	Same as BY4741ΔPEX27	pFARPTS01, pPEX01, pPEX04	This study
MFAOH29	Same as BY4741ΔPEX27	pFARPTS09, pPEX02, pPEX04	This study
MFAOH30	Same as BY4741ΔPEX27	pFARPTS12, pPEX03, pPEX04	This study
MFAOH31	Same as BY4741	pFARPEX,	This study
MFAOH32	Same as BY4741	pFARPEX, pACC1,	This study
MFAOH33	Same as BY4741	pFARPEX, pACC1, pYlACL	This study
MFAOH34	Same as BY4741ΔRPD3	pFARPEX,	This study
MFAOH35	Same as BY4741ΔRPD3	pFARPEX, pACC1,	This study
MFAOH36	Same as BY4741ΔRPD3	pFARPEX, pACC1, pYlACL	This study
MFAOH37	Same as INVSc1	pFARPEX, pACC1	This study
MFAOH38	Same as BY4741	pAdFARPEX	This study
PEXFLUO	Same as BY4741	pPEX14GFP, pFARCHERRY	This study

^*^All the PTS2 signals used the same X5 amino acids sequence: -QSIKD-.
