# Supplementary material for: Pathway Compartmentalization in Peroxisome of Saccharomyces cerevisiae to Produce Versatile Medium Chain Fatty Alcohols
Source: Sci Rep. 2016 May 27;6:26884. doi: 10.1038/srep26884 (PMC4882508; doi:10.1038/srep26884)
Supplement: Supplementary Information [file srep26884-s1.pdf]

Submitted to *Scientific Reports*

Pathway Compartmentalization in Peroxisome of *Saccharomyces cerevisiae* to Produce Versatile Medium Chain Fatty Alcohols

Jiayuan Sheng[†], Joseph Stevens [†], Xueyang Feng[*]

Department of Biological Systems Engineering, Virginia Polytechnic Institute and State University, Blacksburg, VA 24061

[†] Jiayuan Sheng and Joseph Stevens contributed equally to this work.

* To whom correspondence should be addressed. X.F: Phone: (540) 231-2974; E-mail: xueyang@vt.edu.

Figure S1

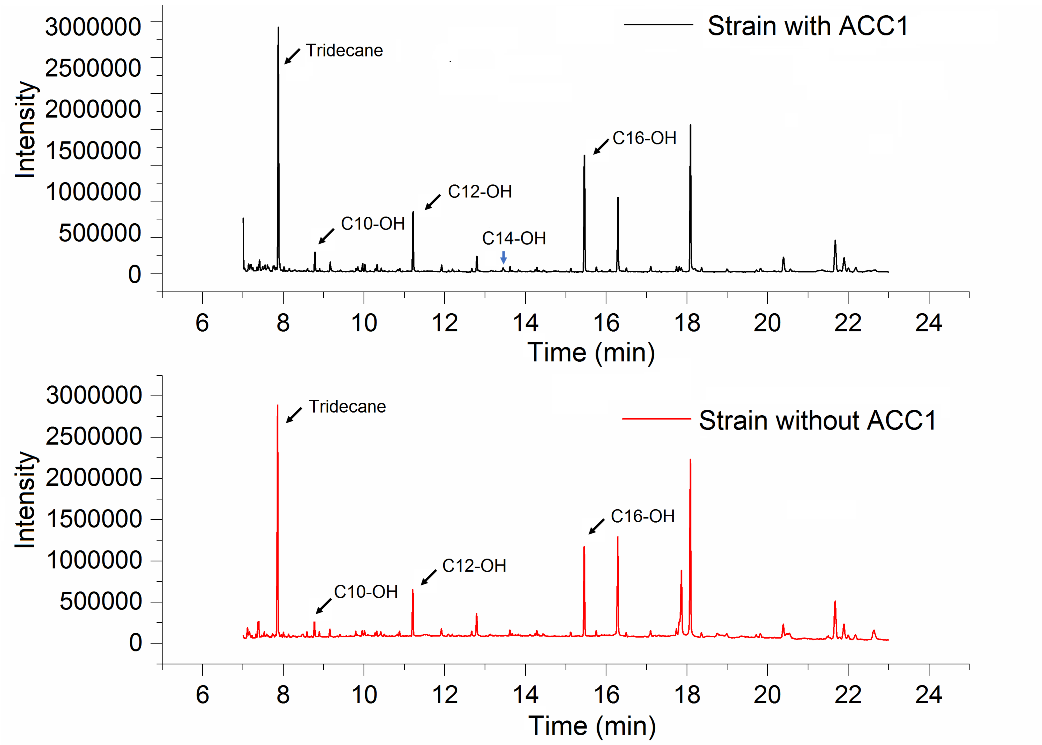

Figure S1: The GC spectrum of fatty alcohols produced by the strains with or without ACC1 overexpressed. Both of the strains were constructed in *S. cerevisiae* BY4741 with TaFAR expressed under constitutive TEF1 promoter and tagged with the PTS2 peptide (KL-QL) and the PEX7 was expressed under constitutive TPI1 promoter.

Figure S2

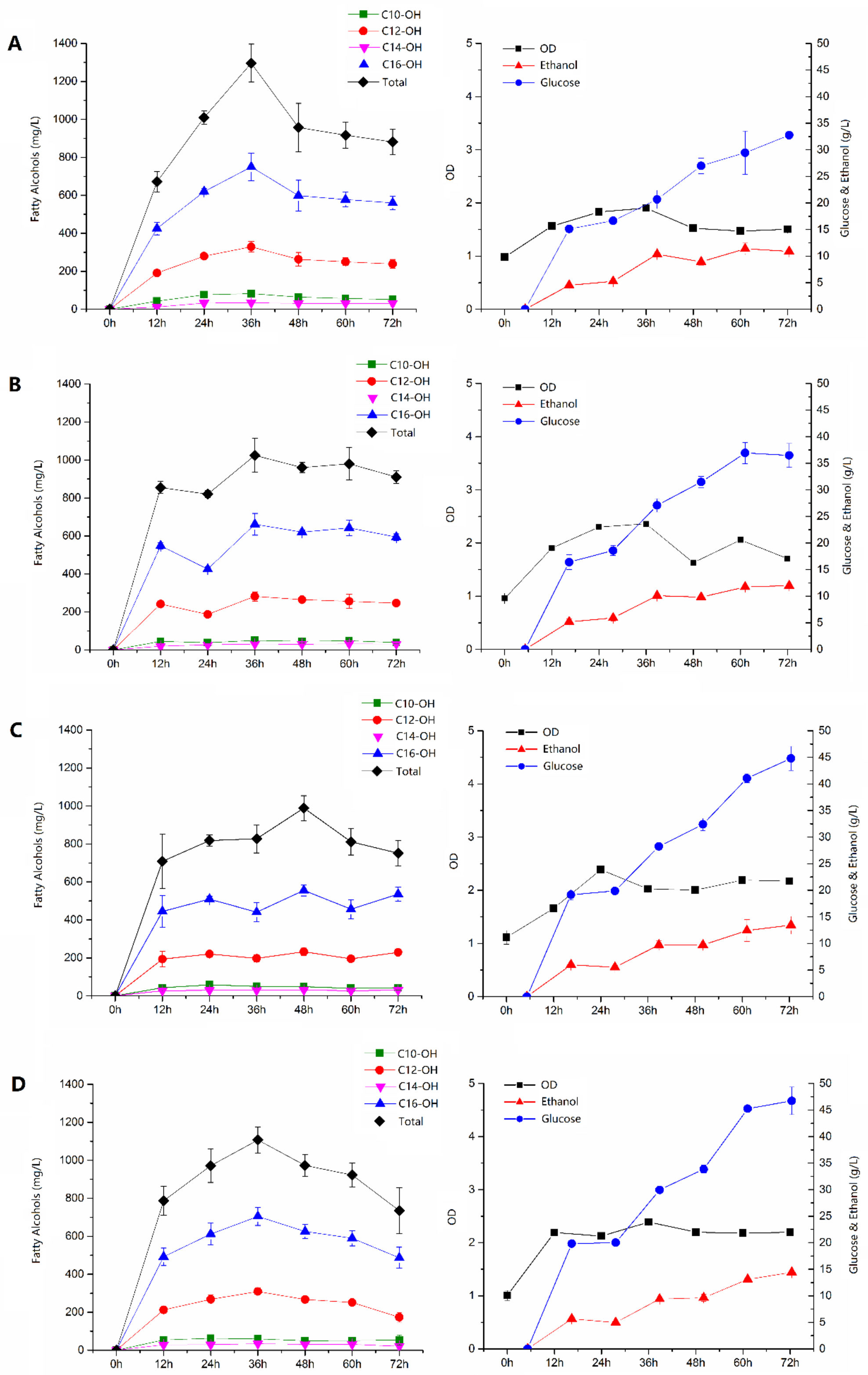

Figure S2. Medium optimization and fed-batch fermentation. (A) Nitrogen limited SC medium with C:N ratio at 50:1; (B) Nitrogen limited SC medium with C:N ratio at 50:1, supplement of 40 mM KCl and 10 mM KOH.; (C) Normal SC medium (control condition with C:N ratio at 3:1); and (D) Normal SC medium with supplement of 40 mM KCl and 10 mM KOH.

Figure S3

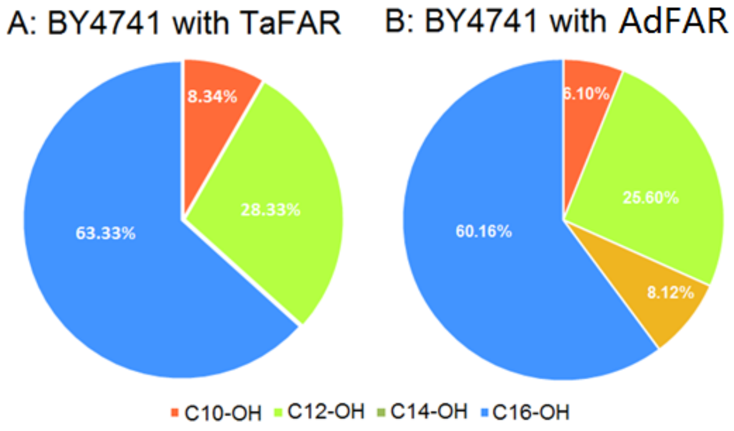

Figure S3: Profiles of fatty alcohols produced by expressing different FAR enzymes in peroxisome. (A) Strain MFAOH31 with TaFAR expressed in peroxisome (8.34% 1-decanol, 28.33% 1-dodecanol and 63.33% 1-hexadecanol); (B) Strain MFAOH38 with AdFAR1 expressed in peroxisome (6.10% 1-decanol, 26.60% 1-dodecanol, 8.12% 1-tetradecanol and 60.16% 1-hexadecanol).

Table S1 The primers used in this study.

Name	Sequence (5'-3')
TEF1p F	AAAGAAAGCATAGCAATCTAATCTAAGTTTTAATTACAAAATGGTTTCCATACCTGAATA
TEF1t R	GCGCAATTAACCCTCACTAAAGGGAACAAAAGCTGGAGCTCGATAGCGCCGATCAAAGTA
TaFAR-SKL R	ATATAAAAGATATGCAACTAGAAAAGTCTTATCAATCTCCTCATAGCTTGGAGTATCTCA
TaFAR-SKF R	ATATAAAAGATATGCAACTAGAAAAGTCTTATCAATCTCCTCAGAACTTGGAGTATCTCA
TaFAR-SFL R	ATATAAAAGATATGCAACTAGAAAAGTCTTATCAATCTCCTCATAGGAAGGAGTATCTCA
TaFAR-SKV R	ATATAAAAGATATGCAACTAGAAAAGTCTTATCAATCTCCTCAAACCTTGGAGTATCTCA
TaFAR-LKL R	ATATAAAAGATATGCAACTAGAAAAGTCTTATCAATCTCCTCATAGCTTTAGGTATCTCA
TaFAR-FKL R	ATATAAAAGATATGCAACTAGAAAAGTCTTATCAATCTCCTCATAGCTTGAAGTATCTCA
TaFAR-SHL R	ATATAAAAGATATGCAACTAGAAAAGTCTTATCAATCTCCTCATAGATGGGAGTATCTCA
TEF1t-SKL F	CTTCAGAGCCTCCAGTACTATGAGATACTCCAAGCTATGAGGAGATTGATAAGACTTTTC
TEF1t-SKF F	CTTCAGAGCCTCCAGTACTATGAGATACTCCAAGTTCTGAGGAGATTGATAAGACTTTTC
TEF1t-SFL F	CTTCAGAGCCTCCAGTACTATGAGATACTCCTTCCTATGAGGAGATTGATAAGACTTTTC
TEF1t-SKV F	CTTCAGAGCCTCCAGTACTATGAGATACTCCAAGGTTTGAGGAGATTGATAAGACTTTTC
TEF1t-LKL F	CTTCAGAGCCTCCAGTACTATGAGATACCTAAAGCTATGAGGAGATTGATAAGACTTTTC
TEF1t-FKL F	CTTCAGAGCCTCCAGTACTATGAGATACTTCAAGCTATGAGGAGATTGATAAGACTTTTC
TEF1t-SHL F	CTTCAGAGCCTCCAGTACTATGAGATACTCCCATCTATGAGGAGATTGATAAGACTTTTC
RV-QL F	AAAGAAAGCATAGCAATCTAATCTAAGTTTTAATTACAAAATGAGTCAACGTGTTCAAAGTATTA AAGATCAACTTGTTTCCATACCTGAATATTAT
KL-QL F	AAAGAAAGCATAGCAATCTAATCTAAGTTTTAATTACAAAATGAGTCAAAAACTTCAAAGTATTA AAGATCAACTTGTTTCCATACCTGAATATTAT
KI-QL F	AAAGAAAGCATAGCAATCTAATCTAAGTTTTAATTACAAAATGAGTCAAAAAATTCAAAGTATTA AAGATCAACTTGTTTCCATACCTGAATATTAT
KV-HL F	AAAGAAAGCATAGCAATCTAATCTAAGTTTTAATTACAAAATGAGTCAAAAAGTTCAAAGTATTA AAGATCATCTTGTTTCCATACCTGAATATTAT
TEF1p RV-QL R	AAAGAAAGCATAGCAATCTAATCTAAGTTTTAATTACAAAATGAGTCAACGTGTTCAAAG
TEF1p KL-QL R	AAAGAAAGCATAGCAATCTAATCTAAGTTTTAATTACAAAATGAGTCAAAAACTTCAAAG
TEF1p KI-QL R	AAAGAAAGCATAGCAATCTAATCTAAGTTTTAATTACAAAATGAGTCAAAAAATTCAAAG
TEF1p KV-HL R	AAAGAAAGCATAGCAATCTAATCTAAGTTTTAATTACAAAATGAGTCAAAAAGTTCAAAG
PEX5 F	AAAGAAAGCATAGCAATCTAATCTAAGTTTTAATTACAAAATGGACGTAGGAAGTTGCTC
PEX5 R	ATATAAAAGATATGCAACTAGAAAAGTCTTATCAATCTCCTTAAAACGAAAATTCTCCTT
TEP1p PEX5 R	CAAGCGGATTATTTCCCACTGAGCAACTTCCTACGTCCATTTTGTAATTAAAACTTAGAT
TEP1t PEX5 F	CATGGACCTGAAAAGATTTAAAGGAGAATTTTCGTTTTAAGGAGATTGATAAGACTTTTC
PEX7 F1	AAAGAAAGCATAGCAATCTAATCTAAGTTTTAATTACAAAATGCTCAGATATCATATGCA
PEX7 R1	ATATAAAAGATATGCAACTAGAAAAGTCTTATCAATCTCCTCAACCTAAGCCGTTCCATA
TEF1p PEX7 R	CACCGTACCCACTAAAACCTTGCATATGATATCTGAGCATTTTGTAATTAAAACTTAGAT
TEF1t PEX7 F	ATGGGATGGAAATTTATTTGTATGGAACGGCTTAGGTTGAGGAGATTGATAAGACTTTTC
TPI1p F	ATTCGATATTGTCGTAACAAATACTTTGATCGGCGCTATCTATATCTAGGAACCCATCAG
TPI1p PEX7 R	CACCGTACCCACTAAAACCTTGCATATGATATCTGAGCATTTTTAGTTTATGTATGTGTT
PEX7 F2	TTAAATCTATAACTACAAAAAACACATACATAAACTAAAAATGCTCAGATATCATATGCA
PEX7 R2	TAACTAATTACATGATATCGACAAAGGAAAAGGGGCCTGTTCAACCTAAGCCGTTCCATA

CYC1t PEX7 F	ATGGGATGGAAATTTATTTGTATGGAACGGCTTAGGTTGAACAGGCCCCTTTTCCTTTGT
CYC1t R	CCACCGCGGTGGCGGCCGCTCTAGAACTAGTGGATCCCCCGCAAATTAAAGCCTTCGAGC
TEF1p PEX3 R	GTCTCTGCAGAAGCGAACGTGATCTTTGATTTGGGGCCATTTTGTAATTAAAACTTAGAT
PEX3 F	AAAGAAAGCATAGCAATCTAATCTAAGTTTTAATTACAAAATGGCCCCAAATCAAAGATC
PEX3 R	ATATAAAAGATATGCAACTAGAAAAGTCTTATCAATCTCCTTAAGGCTTGAAGGAAAACG
TEF1t PEX3 F	CAACTTTGGCGTCTCCAGCTCGTTTTCCTTCAAGCCTTAAGGAGATTGATAAGACTTTTC
TPI1p PEX19 R	ACTCGTTTTCATTCATTACTTCGTGTTGTATGTTTGGCATTTTTAGTTTATGTATGTGTT
PEX19 F	TTAAATCTATAACTACAAAAAACACATACATAAACTAAAAATGCCAAACATACAACACGA
PEX19 R	TAACTAATTACATGATATCGACAAAGGAAAAGGGGCCTGTTTATTGTTGTTTGCAACCGT
CYC1t PEX19 F	CCTTGATAAGGAATTAACCGACGGTTGCAAACAACAATAAACAGGCCCCTTTTCCTTTGT
TEF1p PEX14 R	CGAACAATGCCTTACGATCTTTACTGACCACGTCACTCATTTTGTAATTAAAACTTAGAT
PEX14F	AAAGAAAGCATAGCAATCTAATCTAAGTTTTAATTACAAAATGAGTGACGTGGTCAGTAA
PEX14R	CTTTAGACATACCACCAGATTGAAAATACAAATTTTCACCTGGGATGGAGTCTTCGACCT
GFP F	CTCCATCCCAGGTGAAAATTTGTATTTTCAATCTGGTGGTATGTCTAAAGGTGAAGAATT
GFP R	ATATAAAAGATATGCAACTAGAAAAGTCTTATCAATCTCCTTATTTGTACAATTCATCCA
TEF1t GFP F	TGCTGGTATTACCCATGGTATGGATGAATTGTACAAATAAGGAGATTGATAAGACTTTTC
TaFAR mcherry R	TGCTAACCATACCACCAGATTGAAAATACAAATTTTCACCGTATCTCATAGTACTGGAGG
Cherry F	TATGAGATACGGTGAAAATTTGTATTTTCAATCTGGTGGTATGGTTAGCAAAGGCGAGGA
Cherry R	ATATAAAAGATATGCAACTAGAAAAGTCTTATCAATCTCCTTATAGCTTGGAACCACCAG
TEF1t cherry F	TGAAAATTTGTATTTTCAATCTGGTGGTTCCAAGCTATAAGGAGATTGATAAGACTTTTC
AdFAR F	AAAGAAAGCATAGCAATCTAATCTAAGTTTTAATTACAAAATGTCTCAAAGACTACAAAG
AdFAR R	ATAAAAGATATGCAACTAGAAAAGTCTTATCAATCTCCTTATAGCTTGGAGTAACGCATG
TEF1P_AdFAR R	CCAAATGATCCTTGATACTTTGTAGTCTTTGAGACATTTTGTAATTAAAACTTAGATTAG
TEF1T_AdFAR F	CTTCAGGGCAAGCTCCACCATGCGTTACTCCAAGCTATAAGGAGATTGATAAGACTTTTC
